# mSphere of Influence: Of Mice, Men, and Microbes—How Well Do Experimental Models Recapitulate Human Infection?

**DOI:** 10.1128/mSphere.00228-21

**Published:** 2021-03-31

**Authors:** Chelsie E. Armbruster

**Affiliations:** a Department of Microbiology and Immunology, Jacobs School of Medicine and Biomedical Sciences, Buffalo, New York, USA

**Keywords:** *Escherichia coli*, *Pseudomonas aeruginosa*, RNA-Seq, cystic fibrosis, urinary tract infection

## Abstract

Chelsie Armbruster studies catheter-associated urinary tract infection and the contribution of microbe-microbe interactions to infection progression and severity. In this mSphere of Influence article, she reflects on how two papers, A. E. Frick-Cheng, A. Sintsova, S. N. Smith, M. Krauthammer, et al., mBio 11:e01412-20, 2020, https://doi.org/10.1128/mBio.01412-20, and D. M. Cornforth, F. L. Diggle, J. A. Melvin, J. M. Bomberger, and M. Whiteley, mBio 11:e03042-19, 2020, https://doi.org/10.1128/mBio.03042-19, have impacted her thinking about the bacterial strains and experimental models used to study pathogenesis.

## COMMENTARY

In 1988, Stanley Falkow proposed the idea of “molecular Koch’s postulates” for the emerging field of microbial pathogenesis as a framework for investigating the genetic and molecular basis of pathogenicity (1, 2). One postulate states that inactivation of the gene encoding a suspected virulence factor should reduce pathogenicity or virulence, and addressing this necessitates the use of a suitable infection model. There are countless model systems for assessing microbial pathogenicity and virulence, and numerous pros and cons to be weighed when choosing which model to use for a specific research question. However, there is always the question of whether any model fully recapitulates all of the factors that contribute to establishment of disease in the native host. This issue becomes even more important when studying the pathogenesis of microbes that are typically part of a polymicrobial community, as the pathogenic potential of each microbe as well as the host environment is influenced by the interplay between all constituents. Two recent publications have tackled the question of how well experimental models recapitulate human infection, and they collectively provide a stunning assessment of the similarities and differences in microbial transcriptional profiles during cystic fibrosis and urinary tract infection in humans compared to common model systems (3, 4).

In the first paper, Frick-Cheng et al. used RNA sequencing (RNA-Seq) to compare the transcriptomes of three clinical isolates of uropathogenic Escherichia coli (UPEC) under four conditions: (i) directly from human urinary tract infection (UTI) urine samples, (ii) during experimental infection in a mouse model of UTI, (iii) during growth in human urine *in vitro*, and (iv) during growth in LB broth *in vitro* (4). Principal-component analysis (PCA) of the genes present in all three UPEC isolates demonstrated clear separation between the four transcriptome sets, although a large part of this was driven by expression of ribosomal proteins and may pertain to growth rate. Overall, this impressive study revealed a strong correlation in expression profiles between mouse UTI and human UTI (*r* range of 0.86 to 0.87), with differentially expressed genes largely pointing to differences in nutrient availability and utilization. The authors also made a surprising observation that growth in LB may be a better *in vitro* surrogate for UPEC UTI than growth in human urine, which may again be driven by differences in growth rate.

In the second paper, Cornforth et al. utilized RNA-Seq to compare Pseudomonas aeruginosa transcriptomes from 20 sputum samples collected directly from 19 cystic fibrosis (CF) patients to 67 published P. aeruginosa transcriptomes from various experimental conditions, including three mouse infection models (3). PCA of genes with at least one mapped read in all transcriptomes revealed that the P. aeruginosa transcriptomes from human CF sputum samples were distinct from all of the experimental transcriptomes, including mouse infection studies. The expansive data set allows for assessment of how well each model recapitulates gene expression during human infection, as well as some exciting comparisons such as the metabolic pathways favored by P. aeruginosa during human infection compared to *in vitro* growth or experimental infection. For instance, *in vitro* growth in synthetic CF sputum medium had high accuracy for recapitulating expression of genes pertaining to purines, pyrimidines, nucleosides, and nucleotides, but it did not perform as well as an epithelial cell infection model for mimicking fatty acid and phospholipid metabolism. The authors further demonstrate that combining data from multiple models increases the accuracy of type strain transcriptomes for mimicking human infection, although expression of some “elusive” genes was recapitulated only by using a CF clinical isolate rather than a type strain. Even after combining models or using clinical isolates, a small percentage of gene expression profiles appeared to be specific to human infection samples. The authors therefore speculate that fully recapitulating expression of these genes may require incorporation of a polymicrobial community.

Both of these studies demonstrate the ability of *in vitro* models to accurately recapitulate certain aspects of pathogen gene expression during human infection, while also highlighting nuances of human infection that are challenging to address in experimental models. It is notable that both studies identified differences pertaining to nutrient acquisition and metabolism *in vitro* and *in vivo*, underscoring the critical role of the host nutrient environment for pathogenesis. This is a point that I find particularly important when considering polymicrobial infection, especially for UTI and catheter-associated UTI (CAUTI), since urine represents a nutrient-limited and iron-restricted environment. While my fascination with polymicrobial interactions was initially sparked by interspecies quorum sensing, studies like these have expanded my approach to this subject over the years to include an examination of metabolic cross talk and ways in which the infection environment and polymicrobial milieu influence the metabolic preferences and pathogenic potential of individual microbes.

I initially began investigating the impact of cocolonization on UTI and CAUTI severity as a postdoc in the lab of Harry Mobley, and I chose to focus on two bacterial species that often cocolonize individuals with indwelling urinary catheters (Proteus mirabilis and Providencia stuartii) (5, 6). This was during a time when the Mobley lab was starting to generate transposon mutant libraries in several Gram-negative species for transposon insertion site sequencing (Tn-Seq) approaches, so I set out to utilize Tn-Seq for identification of the core fitness factors of both P. mirabilis and P. stuartii in a mouse model of CAUTI and to further determine the impact of cocolonization on their respective fitness requirements. I had expected to uncover differences pertaining to adhesins, toxins, and more traditional virulence factors given the increased disease severity that occurs during coinfection, but Tn-Seq largely revealed differential requirements for metabolic pathways and nutrient import mechanism (7, 8). At the same time, Carolyn Ibberson was studying the impact of coinfection with P. aeruginosa on Staphylococcus aureus fitness requirements in Marvin Whiteley’s lab and similarly demonstrated a dramatic impact of coinfection on fitness requirements in a mouse model of chronic wound infection, particularly for genes pertaining to energy production and conversion pathways (9). These are just a few examples of the myriad of ways in which polymicrobial interactions influence fitness requirements and metabolic preferences, and the interactions between microbes in various infection models are therefore likely to be highly influenced by culture conditions and the nutrient environment. It will be fascinating to see how well various *in vitro* models of polymicrobial infection recapitulate human infection using the frameworks proposed by Frick-Cheng et al. and Cornforth et al. and what further insights will be gained by integrating transcriptomic analyses with data from other large-scale genetic screens like TraDIS/Tn-Seq.
